# Community-Acquired Severe Clostridium difficile Enteritis Complicated by Metabolic Acidosis and Acute Kidney Injury

**DOI:** 10.7759/cureus.41804

**Published:** 2023-07-13

**Authors:** Saaya Ichiyama, Shunsuke Soma, Katsunori Ito

**Affiliations:** 1 Emergency and Disaster Department, Hirosaki University, Hiorosaki, JPN; 2 Emergency and Critical Care Center, Aomori Prefectural Central Hospital, Aomori, JPN

**Keywords:** ribotype, clostridium difficile infection severity index, septic shock, clostridium difficile infection, community-acquired enteritis

## Abstract

Clostridium difficile (CD) is known to be pathogenic when the balance of intestinal microbiota is disrupted by the administration of broad-spectrum antimicrobial agents. Therefore, CD enteritis is often suspected in cases of hospital-onset diarrhea. There has been a rise in the incidence of community-acquired CD enteritis in recent years in the United States. In this report, we present a case of a 57 year-old-man who was admitted to the emergency department with abdominal distension and dyspnea. The patient suffered from acute renal failure and metabolic acidosis from enteritis. He required mechanical ventilation and continuous renal replacement therapy (CRRT) in the ICU. Analysis of the patient’s stool sample on admission revealed the presence of CD antigens, and the prompt administration of metronidazole led to swift improvement.

No studies have investigated the actual incidence of community-acquired CD enteritis infection in Japan. Since 20% of community-acquired CD enteritis cases have been reported as severe, all cases of community-acquired enteritis should raise concerns for CD enteritis. CD antigen/toxin in the stool should then be determined promptly before administering antibiotics.

## Introduction

Clostridium difficile (CD) enteritis has been reported to cause imbalances in the intestinal microbiota, resulting from the use of broad-spectrum antibiotics, admission to healthcare facilities, and inflammatory bowel disease [1]. In recent years, there has been an increase in the incidence of community-acquired CD enteritis in the United States [2], but its prevalence in Japan is unknown. We discuss a severe case of community-acquired enteritis, where early identification of and treatment modification for CD enteritis led to a favorable outcome. It is critical to distinguish between common bacterial enteritis and CD enteritis because different antimicrobial agents are used for the treatment of these two conditions.

## Case presentation

A 57-year-old man with no history of hospitalization was brought to the emergency department due to symptoms of abdominal distension and dyspnea, and watery diarrhea two days prior. The patient manifested Kussmaul breathing with a respiratory rate of 30 beats/min and SpO_2_ of 98%. His blood pressure and heart rate were within the normal ranges, and the Glasgow Coma Scale (GSC) score was E3V4M6. The patient’s temperature was 35.8 °C and the abdomen was distended but soft. Blood tests revealed severe metabolic acidosis, hepatic and renal dysfunction, dehydration, increased inflammatory markers, and hypoglycemia (Table 1).

**Table 1:** Summary of the laboratory tests and bacterial culture results

Variables	Result	Reference
Biochemistry		
Aspartate aminotransferase (U/L)	241	13 - 30
Alanine aminotransferase (U/L)	132	Oct-42
Creatinine (mg/dl)	2.06	0.46 - 0.79
Blood urea nitrogen (mg/dL)	26	8.0 - 20.0
Sodium (mmol/L)	141	138 - 145
Potassium (mmol/L)	4.9	3.6 - 4.8
Chloride (mmol/L)	86	101 - 108
C-reactive protein (mg/dL)	0.83	0.00 - 0.14
Complete blood count		
White blood cells (/μL)	31,100	3300 - 8600
Red blood cells (×10^6^/μL)	4.84	3.86 - 4.92
Hemoglobin (g/dL)	16.9	11.6 - 14.8
Hematocrit (%)	55.8	35.1 - 44.4
Platelet (×10^4^/μL)	25.1	15.8 - 34.8
Arterial blood gas analysis		
Potential hydrogen (mmHg)	6.73	7.35 - 7.45
Partial pressure of arterial oxygen (mmHg)	67.9	83 - 108
Partial pressure of arterial carbon dioxide (mmHg)	19.1	35 - 48
Bicarbonate ion (mmol/L)	2.4	22.2 - 28.3
Base excisis (mmol/L)	-29.2	-5
Lactate (mmol/L)	29	0.59 - 1.39
Anaerobic culture of stool		
CD antigen	Positive	Negative
CD toxin	Positive	Negative

**Figure 1:**
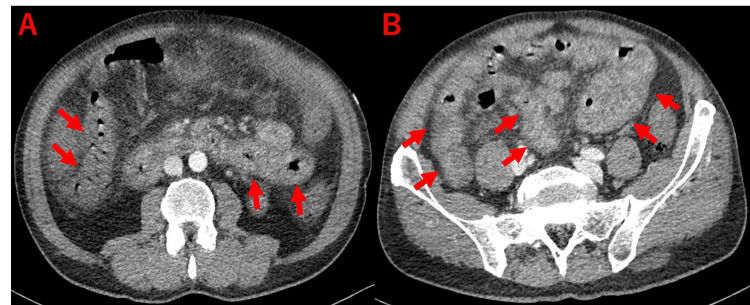
Contrast-enhanced CT scans The arrows demonstrate intestinal edema involving the small to large intestines CT: computed tomography

Contrast-enhanced CT (CECT) scan showed fluid accumulation in the intestinal tract and intestinal edema (Figure 1).

Tests for CD antigens and toxins were conducted according to the recommendations of an experienced nurse based on the smell of the stools. The patient’s stool sample revealed the presence of CD antigens and metronidazole was subsequently administered. Despite aggressive fluid resuscitation, the patient's condition failed to improve and he required mechanical ventilation and continuous renal replacement therapy (CRRT) in the ICU. Diarrhea frequency decreased two days after metronidazole administration. The patient showed rapid improvement in metabolic acidosis and was weaned off of CRRT and ventilator support. The patient was discharged home on the 12th day of disease onset with no diarrhea or other symptoms.

## Discussion

We discuss a case of severe sepsis caused by CD enteritis. Based on the patient’s recent medical history, community-acquired enteritis was suspected. CD enteritis is known to be pathogenic because of the disruption to normal intestinal flora by antimicrobial agents. Our patient was unaware that he had CD enteritis, and a fatal outcome might have occurred if he had been treated as a case of common bacterial enteritis.

CD antigens/toxins are generally evaluated in patients who develop the disease during hospitalization or institutionalization because antibiotics are not readily available in Japanese pharmacies and CD enteritis is often associated with the use of antibiotics. A notable uptick in cases of community-acquired CD enteritis has been reported in the United States [2]. Such cases are typically observed in younger patients with no previous exposure to antibiotics [3], and those with multiple chronic conditions such as heart disease, chronic kidney disease, and inflammatory bowel disease [2]. Our patient had no previously reported risk factors. This may suggest that further studies are needed on the pathogenesis and risk factors of community-acquired CD enteritis.

Our patient manifested severe sepsis requiring ICU care. About 20% of community-acquired CD enteritis cases have been reported as severe [4]. Lungulescu et al. devised a CD infection severity index consisting of four predictors: history of malignancy, white blood cell count at admission >20,000/µL, blood albumin <3.0. mg/dL, and creatinine at admission >1.5-fold the baseline value [5]. A French study by Ogielska et al. indicated that the four predictors of the CD infection severity index have been more prevalent in cases of community-acquired infection [6] and noted the risk of community-acquired CD infection within 2.5 months of using antimicrobial agents, as well as with the use of proton pump inhibitors [6]. After diagnosis and evaluation of the severity of community-acquired CD enteritis infection, oral vancomycin therapy should be promptly started to avoid the overuse of antibiotics [7].

Prevalent strains of CD are known to vary by region. Although the epidemiology of the present case has not been investigated to this degree, our patient was an isolated case, with no outbreak of refractory severe enteritis reported in the area where he resided. Therefore, the hypothesis that this case was caused by a fulminant strain such as ribotype 027 does not hold true.

## Conclusions

Even when there is a suspicion of community-acquired infection in cases of severe enteritis, the management approach differs between CD enteritis and community-acquired enteritis. Consequently, establishing the differentiation between CD enteritis and community-acquired enteritis is imperative. Performing stool culture testing for suspected CD enteritis facilitates the administration of appropriate treatment and promotes favorable outcomes. Epidemiological characteristics of community-acquired CD enteritis in Japan have not been sufficiently investigated, and this remains an issue of grave concern.
